# Refolded scFv Antibody Fragment against Myoglobin Shows Rapid Reaction Kinetics

**DOI:** 10.3390/ijms151223658

**Published:** 2014-12-18

**Authors:** Hyung-Nam Song, Jun-Hyuck Jang, Young-Wan Kim, Dong-Hyung Kim, Sung-Goo Park, Myung Kyu Lee, Se-Hwan Paek, Eui-Jeon Woo

**Affiliations:** 1 Medical Proteomics Research Center, Korea Research Institute of Bioscience & Biotechnology, Daejeon 305-806, Korea; E-Mails: hotdog707@hanmail.net (H.-N.S.); sgpark@kribb.re.kr (S.-G.P.); mklee@kribb.re.kr (M.K.L.); 2 Department of Biotechnology and Bioinformatics, Korea University, Sejong 339-700, Korea; E-Mail: kdh5611@hanmail.net; 3 Department of Food and Biotechnology, Korea University, Sejong 339-700, Korea; E-Mails: jangjunhyuck@naver.com (J.-H.J.); ywankim@korea.ac.kr (Y.-W.K.); 4 Department of Bio-analytical Science, University of Science and Technology, Daejeon 305-333, Korea

**Keywords:** single-chain variable fragment (scFv), premature antibody, myoglobin, acute myocardial infarction

## Abstract

Myoglobin is one of the early biomarkers for acute myocardial infarction. Recently, we have screened an antibody with unique rapid reaction kinetics toward human myoglobin antigen. Antibodies with rapid reaction kinetics are thought to be an early IgG form produced during early stage of *in vivo* immunization. We produced a recombinant scFv fragment for the premature antibody from *Escherichia coli* using refolding technology. The *scFv* gene was constructed by connection of the *V*_H_–*V*_L_ sequence with a (Gly_4_Ser)_3_ linker. The scFv fragment without the pelB leader sequence was expressed at a high level, but the solubility was extremely low. A high concentration of 8 M urea was used for denaturation. The dilution refolding process in the presence of arginine and the redox reagents GSH and GSSH successfully produced a soluble scFv protein. The resultant refolded scFv protein showed association and dissociation values of 9.32 × 10^−4^ M^−1^·s^−1^ and 6.29 × 10^−3^ s^−1^, respectively, with an affinity value exceeding 10^7^ M^−1^ (*k*_on_/*k*_off_), maintaining the original rapid reaction kinetics of the premature antibody. The refolded scFv could provide a platform for protein engineering for the clinical application for diagnosis of heart disease and the development of a continuous biosensor.

## 1. Introduction

Recently, a premature antibody with rapid reaction kinetics was found, and its characteristics have been reported [1,2,3,4,5]. Antibodies with rapid reaction kinetics are thought to be produced during a premature stage of immunization *in vivo*. In the immune system, repetitive exposure to an antigen over an extended period of time gradually induces somatic hypermutation to produce ordinary antibodies that recognize their antigen with a slow dissociation rate and high binding affinity (e.g., 1000-fold), whereas the premature antibody is thought to be generated during an early stage of production. This is referred to as affinity maturation, which consists of the ongoing generation of B-cells receptor (BCR) due to somatic hypermutation and selection of these cells on the basis of affinity. IgM refers to those antibodies that are produced immediately after an exposure to the disease, while IgG refer to a later response. IgM is a temporary antibody that disappears within two or three weeks, which is then replaced by IgG [6]. Premature antibody is thought to belong to early generation of IgG type. Therefore, fewer exposures resulted in the production of antibody that reacts with its binding partner with rapid association and dissociation kinetics [1]. Research on the premature antibody is in an early stage, and the detailed binding mechanism of the rapid dissociation kinetics is currently not well understood.

There is a growing interest in antibodies with rapid reaction kinetics because of their potential application in recycling and continuous measurement systems [7,8,9]. Unlike conventional antibodies with a high affinity interaction, an antibody with rapid reaction kinetics can be used for a recycling system without the requirement of washing or a separate regeneration step. Continuous measurement can be achieved using a rapid reversible antibody immobilized on a label-free sensor to capture the analyte in the sample [2,4,10]. This concept could be used to construct a continuous sensor that can measure fluctuations in a low concentration of biomarkers *in vivo*. This type of continuous immunosensing technique can lead to a novel approach for monitoring and detecting early warning signs of the occurrence of a disorder, which can be applied using a direct online system in the future.

Myoglobin is one of the well-known and early diagnostic biomarkers of acute myocardial infarction (AMI) that can be detected within three hours after the onset of symptoms [11,12]. Myoglobin levels increase in the blood within 2 h after symptom onset of AMI, peak at six to nine hours, and return to normal within 24 h. Numerous application studies have been published, and several recombinant antibody technologies have been reported on the usage of a myoglobin antibody, highlighting its importance as a biomarker and biomolecule for sensor development [13,14,15]. Because AMI is a typical disorder that requires continuous monitoring for early detection of potential life-threatening situations, protein engineering of a premature antibody against the myoglobin antigen could provide a suitable platform for development of a continuous biosensor system. Currently, there is no report on recombinant antibody engineering for the premature antibody and its application.

Recombinant antibody technologies for the handling of key antibody domains constitute an effective tool and have been increasingly used as alternatives to monoclonal Abs in medical diagnostic and therapeutic applications [16,17,18]. One of the most popular types of recombinant antibodies is the single-chain variable fragment (scFv) because it has been successfully modified into a number of different antibody formats and is easily expressed by several expression systems [19,20,21]. The scFvs contain the complete antigen-binding site, which includes the variable heavy (*V*_H_) and variable light (*V*_L_) domains of an antibody, which are able to bind selectively to a specific antigen. The *V*_H_ and *V*_L_ domains can be linked by one of various flexible linkers (scFv), by a disulfide bond (dsFv) or by both (sc-dsFv) [22,23]. However, the insoluble inclusion body formation of scFvs expressed in *Escherichia coli* is a significant problem [24,25]; therefore, a variety of methods for refolding proteins have been developed [26,27]. In this study, we expressed the insoluble scFv derived from the premature antibody against myoglobin in *E. coli*. We refolded the denatured protein in the presence of additives, such as arginine and a reducing agent, and the resultant scFv fragment retained its original features, such as rapid and reversible kinetics.

## 2. Results

### 2.1. Construction and Expression of 2-7ds scFv for the E. coli System

**Figure 1:**
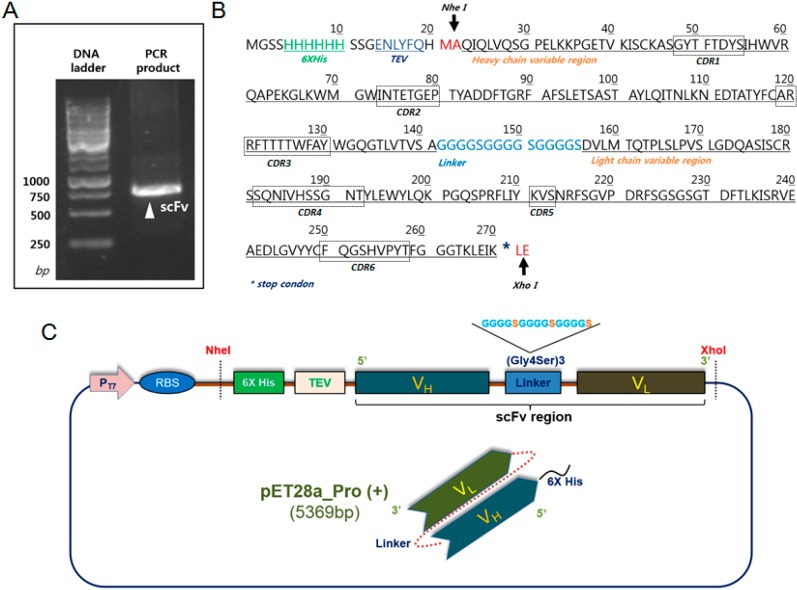
Construction of the premature scFv for protein expression in *E. coli*. The sequence of the premature scFv with a hexa-histidine tag and a TEV sequence in the *N*-terminus was shown with a (Gly_4_Ser)_3_ linker between the variable heavy (*V*_H_) and variable light (*V*_L_) regions. (**A**) The insert of the *scFv* genes was amplified using PCR; (**B**) The NheI (5') and XhoI (3') restriction enzymes were used for the subcloning; and (**C**) Schematic diagram of the vector plasmid for *E. coli* expression.

**Figure 2:**
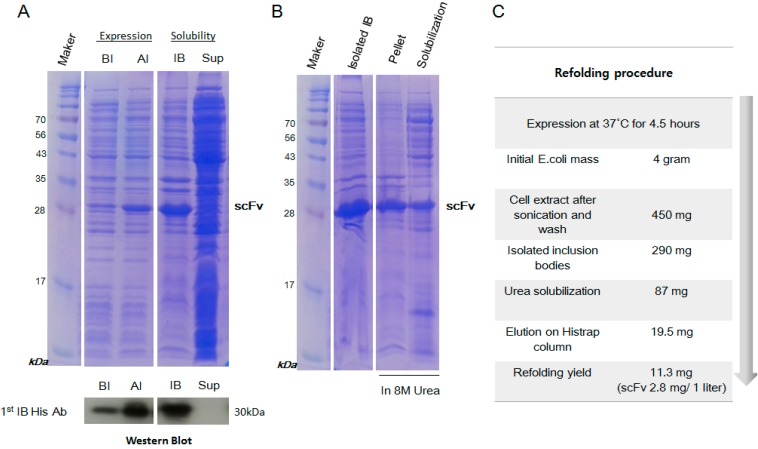
The expression, isolation and solubilization of the scFv protein from *E. coli*. (**A**) The recombinant scFv showed high expression after IPTG induction (AI), corresponding to 30 kDa in SDS-PAGE (**top**) and Western blot analysis (**bottom**) compared with pre-induction (BI); (**B**) SDS-PAGE analysis of the inclusion bodies (IB). The pellet and the solubilized scFv using 8 M urea were separated by centrifugation; and (**C**) Schematic diagram of the scFv refolding steps.

The *scFv* gene was constructed from the monoclonal antibody of two- to seven-days-old hybridoma cell clones immunized using myoglobin antigen (Figure 1A). The DNA plasmid with a hexa-histidine tag and a TEV cleavage site at the *N*-terminus was constructed in the expression vector of pET28a_pro with the (Gly_4_Ser_1_)_3_ flexible linker between the heavy chain and the light chain of the variable regions. The pelB leader sequence, which directs the protein to the bacterial periplasm in ordinary bacterial expression of scFv for correct folding, was removed in this system because the initial expression and solubility was too low to detect (Figure 1B). The expression level of scFv was high on the SDS-PAGE gel compared with the protein with the pelB sequence. We found that the recombinant scFv fragment was mostly insoluble when located in the inclusion body (Figure 2A).

### 2.2. Inclusion Body Isolation and Solubilization of the Recombinant Protein scFv

In the inclusion body, many contaminant proteins were mixed with the target scFv protein. For purification of the inclusion body, the pellet was washed with DTT, which provides a reducing environment, and with Triton X-100 detergent, which solubilizes membrane debris. The inclusion body was successfully solubilized after it was denatured using 8 M urea for 24 h. We found that 13.8% of scFv in inclusion body was solubilized by 8 M urea (Figure 2B). To obtain a high yield of scFv solubilization, it was essential to dissolve the inclusion bodies in an ample volume of the solubilization buffer (1 mL/20–40 mg) in the presence of 8 M urea. Additional sonication processes were also effective for the complete removal of the pellet mass, whereas prolonged incubation resulted in higher solubilization.

### 2.3. Purification and Refolding of scFvs by Rapid Dilution

**Figure 3:**
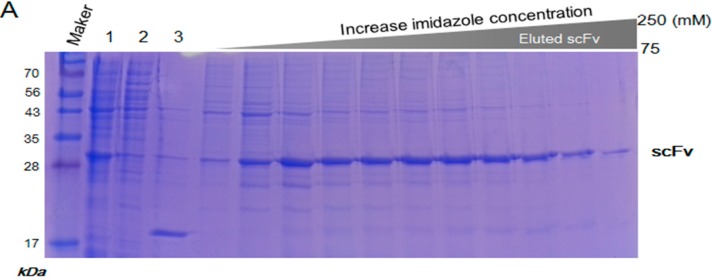

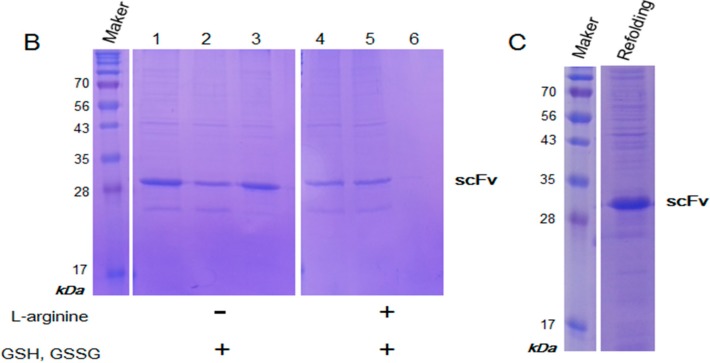
Purification and refolding of the scFv antibody fragment. (**A**) Solubilized scFv was applied to a Ni-column and eluted by a gradient of 250 mM imidazole (lane 1, before column; lane 2, unbound; lane 3, wash); (**B**) The scFv fragment was refolded in the presence of 440 mM l-arginine and the redox reagents, 2 mM GSH and 0.2 mM GSSH (lanes 1 and 4, before refolding sample; lanes 2 and 5, the supernatant after refolding; lanes 3 and 6, the pellet after refolding); and (**C**) The final refolded scFv antibody fragment.

**Figure 4:**
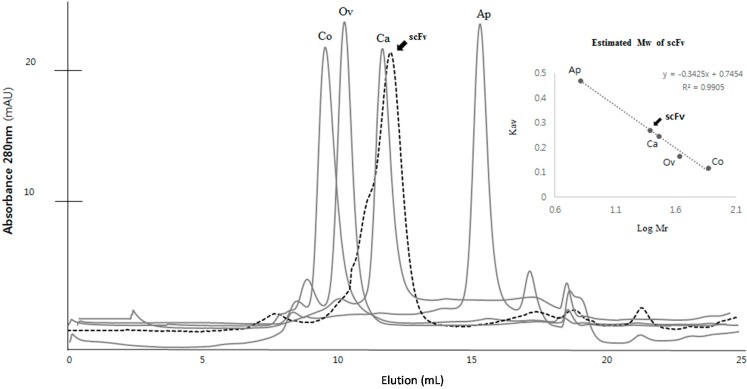
Molecular mass of scFv using a gel filtration column. Molecular weights for standard proteins are as follows: Conalbumin, 75 kDa, 9.55 mL; ovalbumin, 43 kDa, 10.31 mL; Carbonic anhydrase, 29 kDa, 11.65 mL; Aprotinin, 6.5 kDa, 15.33 mL; (gel filtration calibration kit LMW and HMW, GE Healthcare, Piscataway, NJ, USA) The scFv fragment, indicated by a black arrow, with the expected molecular weight of 29 kDa was eluted at the elution volume of 12.04 mL close to the carbonic anhydrase. The gel-phase distribution coefficient (*K*_av_) was calculated from: *K*_av_ = (*V*_e_ − *V*_o_)/(*V*_c_ − *V*_o_) where *V*_e_ is elution volume, *V*_o_ (7.61 mL) is void volume (determined using blue dextran), and *V*_c_ (24 mL) is column volume.

**Figure 5:**
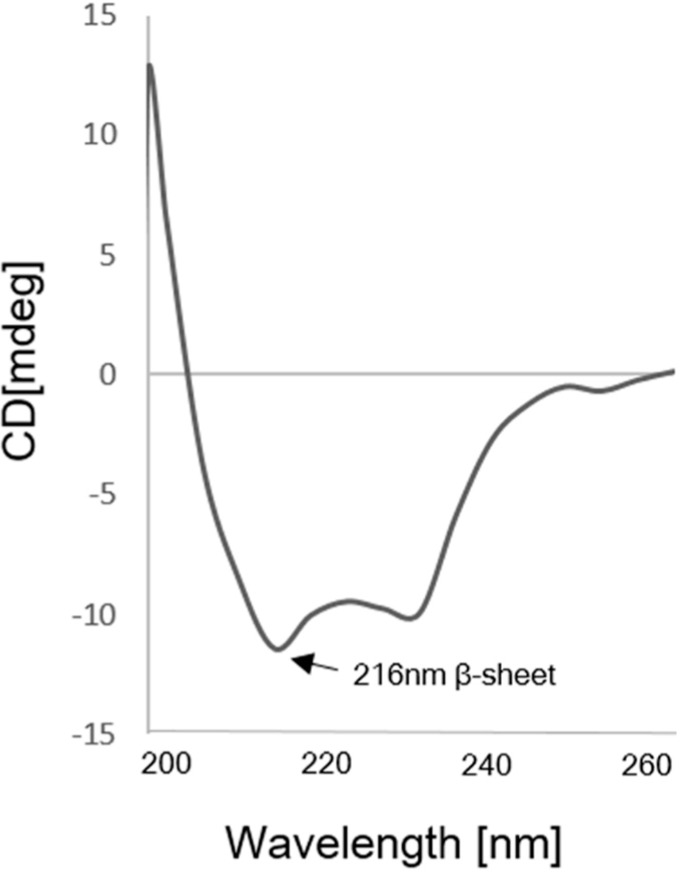
Far-UV circular dichroism (CD) spectra for the refolded scFv. The scFv fragment (0.5 μg/mL) dissolved in 50 mM Tris pH 7.5, 100 mM NaCl and 5% glycerol was analyzed at room temperature.

The solubilized scFv was purified using a nickel chelate column, and the eluted scFv was directly subjected to a refolding step (Figure 3A). It was clearly observed that the concentration of the initial protein samples was an important factor for the refolding of scFv. A high protein concentration (greater than approximately 10 mg/mL) caused significant aggregation upon denaturation of scFv, whereas a low concentration of scFv (approximately 3 mg/mL) showed a good refolding yield. Because the reducing environment inside the *E. coli* system is inappropriate for the folding of scFv, redox reagents, such as reduced glutathione GSH and oxidized glutathione GSSH, were added for optimal folding of scFv *in vitro*. In the refolding step, the proper level of GSH and GSSH was an important factor, and the refolding buffer containing 2 mM of GSH and 0.2 mM of GSSH in a 10:1 ratio provided a good refolding yield in this study. To avoid any unnecessary reduction of the disulfide linkage in the cysteine residues, traces of any reducing agents, such as β-mercaptoethanol or DDT, were removed in subsequent buffers. l-arginine was the key element for minimal aggregation of the denatured protein and for an increase in the solubility of the scFv fragment. When the denatured protein was diluted in a 20-fold refolding buffer containing 440 mM l-arginine, a good solubility level of the refolded protein was attained. The addition of l-arginine increased the solubility by 53.6% (2.15-fold) compared with the absence of l-arginine (Figure 3B). The purity of refolded scFv was analyzed more than 55% in the soluble fraction by SDS-PAGE (Figure 3C). To increase stability of the refolded scFv, the refolding process was maintained at a low temperature (4 °C) because a significant amount of aggregation occurred during refolding at room temperature (26 °C). Finally, the refolded scFvs were separated from the aggregated proteins and the low molecule contaminants using size exclusion chromatography (SEC). The calculated size of the scFv by SEC was approximately 28–29 kDa, which according to SDS-PAGE, showed a single purified band of protein (Figure 4). The structural feature of scFv was verified through secondary structure analysis using circular dichroism (CD). From the CD spectra analysis, the secondary structure of scFv was estimated to consist of 44.8% β-sheet, 0.9% α-helix, and 54.3% random structure, which is in good agreement with the secondary structure prediction based on the amino acid sequence (Figure 5).

### 2.4. Kinetics Assay with Myoglobin Antigen

**Figure 6:**
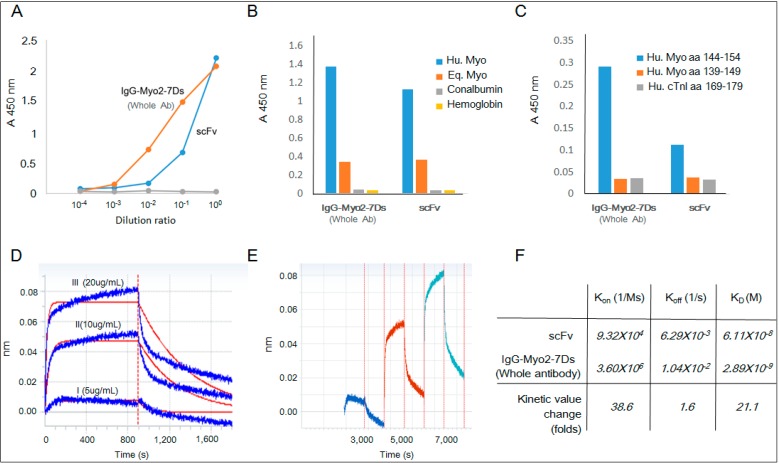
Binding analysis of the refolded scFv fragment. (**A**) Enzyme-linked immunosorbent assay (ELISA) analysis for the binding of scFv on the human myoglobin coated surface. Serial dilutions (×1/10) were made for the premature antibody and the refolded fragment with an initial concentration of 0.1 and 30 μg/mL, respectively; (**B**) Comparative ELISA analysis of the refolded scFv and the whole antibody for human myoglobin, equine myoglobin, conalbumin and hemoglobin (scFv 1.9 μg/mL, IgG2-7Ds 0.5 μg/mL); (**C**) Comparative ELISA analysis of the refolded scFv and the whole antibody for synthetic peptides of human myoglobin and human cardiac troponin I (scFv 1.9 μg/mL, IgG2-7Ds 0.5 μg/mL); (**D**) The binding pattern was analyzed by an Octet-RED immunosensor; (**E**) The binding pattern was analyzed using increment of scFv in a continuous manner; and (**F**) The comparison of the kinetic parameter values for the parent premature antibody and the refolded scFv was performed using the Data Analysis 7.0 program.

We tested whether the refolded scFv has a binding activity to human myoglobin antigen as compared to the whole parental antibody. The antibody binding was investigated using the enzyme-linked immunosorbent assay (ELISA). The refolded scFv showed a relatively weaker interaction with human myoglobin at a low concentration. However, it showed a comparable affinity value (optical density, (OD)) to the parent premature antibody at a higher concentration (Figure 6A). The refolded scFv showed binding affinity to both human myoglobin and equine myoglobin whereas it did not bind to other proteins, such as hemoglobin or conalbumin, being consistent with the binding pattern of the parental antibody. Despite the high sequence similarity between myoglobins, the purified scFv exhibited a significantly higher binding affinity to human myoglobin than to equine myoglobin, indicating its specificity toward human antigen (Figure 6B). Recently we identified the epitope site of the premature antibody to be the *C*-terminal region of the myoglobin antigen [28]. Consistently, the refolded scFv showed binding absorbance to the *C*-terminal region of the myoglobin peptide, aa 144–154, with negligible binding to other peptides (Figure 6C). To further test the binding affinity, a label-free sensor system was used to measure the association and dissociation kinetics against human myoglobin coated on the APS sensor tip surfaces. The result showed effective binding between the refolded scFv and the target antigen, whereas it did not bind the BSA of the negative control. The assay in the Octet system showed an increased intensity with an increase in the scFvs concentration with a repetitive binding pattern in the continuous binding analysis (Figure 6D,E). The measured values of the association rate constant (*k*_on_) and the dissociation rate constant (*k*_off_) for the refolded scFv were 9.32 × 10^4^ M^−1^·s^−1^ and 6.29 × 10^−3^ s^−1^, respectively, with an overall affinity value (*K*_D_ = *k*_off_/*k*_on_) of 10^−8^ M (Figure 6E). Compared with the intact premature antibody, the kinetics data showed that the association rate decreased 38.6-fold from 3.6 × 10^6^ to 9.32 × 10^4^ with a dissociation value showing a 1.6-fold reduction, yielding an overall decrease in the affinity by 23.3-fold [1]. Despite the reduction of the association value, the refolded scFv showed a significantly high dissociation value, maintaining the same unique binding patterns of rapid kinetics of the parent antibody.

## 3. Discussion

In this study, we successfully refolded premature antibody from *E. coli* inclusion bodies using the refolding buffer containing l-arginine. The l-arginine molecule has been known to be a component that minimizes self-aggregation of denatured proteins by increasing their solubility during the refolding process [29,30,31]. In our study, the scFvs in the refolding buffer without l-arginine aggregated noticeably, whereas addition of l-arginine showed a significant increase in the refolding yield. Protein homology modeling based on previous antibody structures suggests that 29.4% of hydrophobic residues, such as I, V, W, Y, F, in the CDR region are exposed on the surface. These structural and sequential elements may have caused the aggregation during purification at a high concentration, even in the presence of 10% glycerol. This result clearly showed that l-arginine stabilized the exposed hydrophobic areas of the recombinant protein and suppressed aggregation of a partially folded intermediate during the refolding procedure. For correct refolding, the redox components GSH and GSSG were supplied in addition to l-arginine in this study. In general, when the native target protein contains disulfides, the redox reagents provide a favorable condition for folding because the rates and yields of the refolding reactions are highly dependent on the redox condition [32], whereas the optimal redox conditions differ for each protein and each set of buffer conditions. Redox reagents reduce non-native protein–protein disulfides that hold the protein in an incompletely oxidized form. In addition, an oxidant, such as GSSG, is essential for disulfide bond formation, although excess GSSG can cause free sulfhydryls of the protein to form disulfides with glutathione that consequently inhibit intramolecular disulfide linkage formation [33].

Using refolded protein partitioned in the inclusion body, we produced an active scFv fragment that could be useful in research and clinical settings due to the modifiable pharmacokinetic properties compared with the parent whole antibodies. Due to their relatively small molecular weight, scFv proteins can be effective in tissue penetration with the relative ease of mass-producing them at a low cost. Here, we successfully refolded the recombinant scFv protein, retaining the original rapid kinetics of the parent premature antibody. The resultant scFv showed a relatively weak association rate with the myoglobin antigen compared with the parental whole antibody. We assume that this result is partly due to the monovalent binding property of scFv [34,35] and partly due to the altered detection method in which the additional wash step was used for detection of the histidine tag at the *N*-terminus of the recombinant antibody. Given the monovalent binding property of scFv and the altered secondary antibody for ELISA detection, the recombinant scFv protein appears to retain a comparable level of rapid kinetics with the parent antibody. Although they have a lower binding affinity, these fragments can be genetically modified to enhance desirable properties in multivalency and high target retention. For example, the scFv can be used in dimers, diabodies, IgG, or multivalent molecules to increase the avidity of low affinity monomers to target antigens. The mechanism of the rapid binding kinetics of the premature antibody remains unknown. An additional biochemical study using the refolded scFv retaining the same binding characteristics of the parent antibody would assist in the comprehension of the molecular mechanism of the unique binding pattern of the premature antibody and the development of an engineered antibody that can be used in a continuous detection system for AMI patients.

## 4. Experimental Section

### 4.1. Plasmid Construction for the Expression of Recombinant scFv

Hybridoma Myo2−7Ds cells were prepared as previously described [1]. The total RNA isolated from hybridoma cells using the TRIzol reagent (Invitrogen, Carlsbad, CA, USA) was used as the template for the synthesis of complementary DNA (cDNA) [36]. A GeneRacer kit (Invitrogen) was used for the cDNA preparation according to the procedures recommended by the manufacturer. The gene fragments for the variable regions of *V*_H_ and *V*_L_ were amplified using the cDNA as the template, and the assembled full gene fragment for scFv-Myo2-7Ds with a (GGGGS) × 3 linker was constructed. The scFv sequence was amplified using primers scFv-Myo-NheI Fw: 5'-GCGCGCTAGCGATGTTTTGATGACCCAA-3' and scFv-Myo-XhoI Rev: 5'-AAGCTGGAAATAAAATAGCTCGAGGCGC-3' by polymerase chain reaction. The *scFv* gene fragment was cloned in pET-28a (+) into the NheI/XhoI restriction sites, and the resulting plasmid was designated as pET28a-scFv-Myo2-7Ds6×H. The thrombin cleavage site of pET28a (+) was replaced with the TEV cleavage site, and the vector system was composed of a 6× His tag at the *N*-terminus with kanamycin tolerance for bacterial growth.

### 4.2. Expression and Isolation of scFv from Inclusion Bodies

The scFv expression vector was transformed using *E. coli* BL21/DE3/RIL competent cells, which were incubated with shaking at 37 °C in Luria Broth (LB) medium supplemented with 30 μg/mL kanamycin. When the OD (optical density) of the cell growth reached 0.6, the induction of scFv expression by the addition of 1 mM IPTG was performed over 4 h 30 min at 30 °C at 10× *g*. The cells were then collected by centrifugation for 30 min at 4615× *g* and stored at −20 °C. The cell pellet (4 g) was thawed at room temperature and subjected to sonication (1 s on/off, 60% amplitude for 2 min; repeated 3 times) in lysis buffer 100 mL containing phosphate-buffered saline (PBS: 137 mM NaCl, 27 mM KCl, 100 mM Na_2_HPO_4_, 20 mM KH_2_PO_4_, pH approximately 7.4), 1 mM PMSF and a protein inhibitor cocktail tablet. The cell lysate was incubated for 30 min on ice and then centrifuged at 15,000× *g* for 15 min at 4 °C to pellet the insoluble material (inclusion bodies). The pellet was suspended in a 100 mL buffer of 50 mM Tris, 10 mM EDTA, 5 mM DTT, 2% Triton X-100, and 500 mM NaCl adjusted to pH 7.5, and the solution was vortexed to ensure that the pellet was completely suspended. The suspension was centrifuged at 15,000× *g* for 15 min at 4 °C to pellet the inclusion bodies (the isolation wash step, repeated twice). The pellet was resuspended in 100 mL buffer of 50 mM Tris and 10 mM EDTA adjusted to pH 7.5, and the suspension was transferred to a centrifuge tube of known weight and centrifuged at 15,000× *g* for 15 min at 4 °C to pellet the inclusion bodies.

### 4.3. Purification and Refolding of scFv

The purified inclusion bodies pellet (2.5 g) was solubilized in 120 mL of 50 mM Tris, pH 7.5 buffer with 8 M urea at 4 °C overnight. After centrifugation at 15,000× *g* for 50 min, the supernatant containing the solubilized scFv was purified using a 25 to 500 mM linear imidazole gradient in 50 mM Tris, 300 mM NaCl, 6 M urea and 1 mM PMSF, pH 7.5 on a Histrap HP 5 mL column using the AKTA purifier (GE Healthcare). The denatured scFv in 6 M urea was refolded using the previously described technique [37,38]. The purified scFv solution was rapidly diluted in 19 volumes of refolding buffer (55 mM Tris, 21 mM NaCl, 0.88 mM KCl, 1 mM EDTA, 2 mM GSH and 0.2 mM GSSG, pH 8.2) at 4 °C for 24 h. The soluble scFv was collected by centrifugation at 15,000× *g* for 30 min at 4 °C to remove the aggregated scFv, dialyzed in TBS (50 mM Tris and 100 mM NaCl, pH 7.5), and then concentrated using a Centricon microconcentrator (Vivaspin 10K MWCO, Sartorius, Weender Landstr, Germany). The concentrate was applied to a Superdex 75 10/300 GL column (prep grade, GE Healthcare) equilibrated with 50 mM Tris, 100 mM NaCl, and 5% glycerol, pH 7.5 and eluted in the downward flow mode at a flow rate of 0.4 mL/min. The fractions were assayed for A_280_ and analyzed for protein contents by SDS-PAGE. The scFv peak fractions were pooled and stored at 2–8 °C. Standard marker proteins (Gel filtration calibration kit LMW, GE Healthcare) were used for molecular weight of the protein in solution.

### 4.4. Circular Dichroism Analysis of scFv

CD spectroscopy was performed on a Jasco J-815 spectropolarimeter (Jasco, Tokyo, Japan). The spectra were recorded using a 1-mm path length cell (equilibrated at room temperature) in a wavelength range from 190 to 260 nm with 1-nm resolution at 50 nm/min scanning speed and a 1 s response time. A concentration of 0.5 mg/mL scFv was dissolved in 50 mM Tris, 100 mM NaCl, and 5% glycerol.

### 4.5. Enzyme-Linked Immunosorbent Assay

The binding activity of refolded scFv was determined by ELISA. 100 μL of the antigen (human myoglobin; 5 μg/mL) dissolved in carbonate buffer (15 mM Na_2_CO_3_, 35 mM NaHCO_3_, 0.2 g/L NaN_3_; pH 9.6) was coated onto each well of a 96-well microarray plate (SPL Life Sciences, Seoul, Korea) at 4 °C for 16 h. After washing with PBS-T (0.05% Tween-20 in PBS) three times, the coated antigens were blocked with 200 μL/well of blocking buffer (10 mg/mL bovine serum albumin in PBST) for 1 h at 37 °C. Serially diluted refolded protein and premature antibody were added to the coated each wells and allowed to bind for 1 h at 37 °C. After washing, 100 μL of 1:2500 diluted anti-6× His tag antibody (Abcam, Cambridge, MA, USA) dissolved in antibody dilution buffer (3% BSA in PBST) was added to each well and allowed to react for 1 h. After washing, 100 μL of 1:5000 diluted goat-anti-Mouse in secondary-HRP conjugated antibody dissolved in antibody dilution buffer was added to each well and allowed to react for 1 h. After washing, 100 μL of a TMB peroxidase substrate system (BD, Franklin Lakes, NJ, USA) was added to each well for detection and 50 μL of 1 N H_2_SO_4_ was added as stopping reagent. The signals were quantified by measuring absorbance at 450 nm by using a spectrophotometer (Bio-Rad, Hercules, CA, USA). For comparison, BSA was coated onto the plate, and all the steps as previously described were performed.

### 4.6. Reaction Kinetics of Myoglobin Binding to scFv

The association and dissociation kinetics of myoglobin and scFv were analyzed using the label-free sensor system, Octet Red (ForteBio, Menlo Park, CA, USA), according to the manufacturer’s protocol. Human myoglobin (5 μg/mL) in phosphate-buffered saline (PBS, pH 7.4) was immobilized on the surfaces of an APS disposable sensor tip at 30 °C for 900 s, and the residual surfaces were blocked by 0.5% casein in PBS (casein–PBS). The myoglobin-immobilized sensor was dipped into the antibody solution (1 μg/mL) in casein–PBS to analyze the association reaction and was subsequently returned to the casein-PBS solution to analyze the dissociation reaction. The association and dissociation kinetic curves were obtained by subtracting the data obtained in the absence of myoglobin using the Data Analysis 7 program (ForteBio). The rate constants for association (*k*_on_) and dissociation (*k*_off_) were obtained by regression analysis using the Data Analysis 7 program, and the equilibrium association constant (*K*_A_) was calculated using the equation *K*_A_ = *k*_on_/*k*_off_.

## 5. Conclusions

Research on premature antibodies is still in its infancy and the detailed binding mechanism of the rapid reaction is not known yet. Here, we produced the scFv of the premature antibody for human myoglobin antigen, purified it from inclusion body and successfully refolded to active form using reagents including arginine and DTT. The refolded scFv retains the unique rapid reaction binding activity toward myoglobin. The active scFv fragment could be used for research in understanding the binding mechanism of the rapid dissociation and the development of an engineered antibody in continuous system for AMI patients.
